# Mutants for *Drosophila* Isocitrate Dehydrogenase 3b Are Defective in Mitochondrial Function and Larval Cell Death

**DOI:** 10.1534/g3.116.037366

**Published:** 2017-01-17

**Authors:** Dianne M. Duncan, Paula Kiefel, Ian Duncan

**Affiliations:** Department of Biology, Washington University, St. Louis, Missouri 63130

**Keywords:** Idh3b, isocitrate dehydrogenase, E93, apoptosis, mitochondria, autophagy

## Abstract

The death of larval salivary gland cells during metamorphosis in *Drosophila melanogaster* has been a key system for studying steroid controlled programmed cell death. This death is induced by a pulse of the steroid hormone ecdysone that takes place at the end of the prepupal period. For many years, it has been thought that the ecdysone direct response gene *Eip93F* (*E93*) plays a critical role in initiating salivary gland cell death. This conclusion was based largely on the finding that the three “type” alleles of *E93* cause a near-complete block in salivary gland cell death. Here, we show that these three mutations are in fact allelic to *Idh3b*, a nearby gene that encodes the β subunit of isocitrate dehydrogenase 3, a mitochondrial enzyme of the tricarboxylic acid (TCA) cycle. The strongest of the *Idh3b* alleles appears to cause a near-complete block in oxidative phosphorylation, as mitochondria are depolarized in mutant larvae, and development arrests early during cleavage in embryos from homozygous-mutant germline mothers. *Idh3b*-mutant larval salivary gland cells fail to undergo mitochondrial fragmentation, which normally precedes the death of these cells, and do not initiate autophagy, an early step in the cell death program. These observations suggest a close relationship between the TCA cycle and the initiation of larval cell death. In normal development, tagged Idh3b is released from salivary gland mitochondria during their fragmentation, suggesting that Idh3b may be an apoptogenic factor that functions much like released cytochrome *c* in mammalian cells.

The death of larval salivary glands during metamorphosis in *Drosophila* is an important model system for understanding the control of programmed cell death by steroid hormones [for review, see Yin and Thummel (2005)]. Salivary gland cell death shares many features with apoptosis, including hallmark events such as DNA fragmentation, mitochondrial fragmentation (Goyal *et al.* 2007), and exposure of phosphatidylserine on the outer leaflet of the plasma membrane (Lee and Baehrecke 2001). However, unlike apoptosis, in which dead or dying cells are engulfed and degraded by phagocytes, salivary gland cell death is autophagic, and involves degradation of cellular components within autolysosomes [Lee and Baehrecke (2001); reviewed in Baehrecke (2003); Tracy and Baehrecke (2013)]. The key initiating event in salivary gland death is expression of the cell death activators reaper (rpr) and head involution defective (hid). These activators interfere with the activity of the apoptosis inhibitor, diap1, which allows dimerization and activation of the initiator caspase dronc, which then cleaves and activates the downstream execution caspase drice, which, in turn, cleaves additional substrates in the cell death cascade.

The histolysis of larval salivary glands is triggered by the prepupal pulse of the steroid hormone ecdysone. Some early events in the death process are clear: transcription of the cell death activator rpr is activated directly by the ecdysone-receptor complex, with this activation being enhanced by the direct response gene Broad-Complex (BR-C) (Jiang *et al.* 2000). The ecdysone-receptor complex also directly induces expression of the initiator caspase dronc (Cakouros *et al.* 2004). In contrast, *hid* is not regulated directly by the ecdysone-receptor complex, but is activated by the immediate response genes *BR-C* and *E74A* (Jiang *et al.* 2000). This activation of *rpr*, *dronc*, and *hid*, as well as the partial repression of the apoptosis inhibitor *diap1* by CBP (Yin *et al.* 2007), are key events in the initiation of salivary gland histolysis. To understand better the events of salivary gland cell death, large-scale genetic (Wang *et al.* 2008) and molecular (Gorski *et al.* 2003; Lee *et al.* 2003; Martin *et al.* 2007; McPhee *et al.* 2013) screens have been conducted to identify additional genes that mediate the death response. The genes identified encode functionally diverse products, including an RNA helicase (Ihry *et al.* 2012), chromatin remodelers (Ihry and Bashirullah 2014), an enzyme of the TCA cycle (Wang *et al.* 2010), subunits of the mediator complex (Wang *et al.* 2010), subunits of the cop9 signalsome (McPhee *et al.* 2013), several autophagy (Atg) proteins (Berry and Baehrecke 2007), and several proteins of unknown function. The roles of these proteins in salivary gland cell death, and how these roles are coordinated, are, for the most part, poorly understood.

The ecdysone primary response gene *Eip93F* (*E93*) has been thought to play a key role in initiating salivary gland cell death. In their original characterization of *E93*, Lee *et al.* (2000) set out to identify loss-of-function alleles of the locus by screening for new lethal mutations that fail to complement a deficiency that includes the *E93* locus. One of the complementation groups identified became the focus of attention, as its three alleles cause lethality early in metamorphosis, coinciding with the beginning of *E93* transcription. This group was assigned to *E93* when it was found that one of its alleles (“*E93^1^*”) is associated with a nonsense mutation at codon 995 of the E93 A isoform, near the 3′ end of the *E93* coding sequence. The other two alleles in this complementation group (“*E93^2^*” and “*E93^3^*”) do not show changes in the *E93* coding sequence, but were assumed to have changes elsewhere in the gene. Since this early work, “*E93^1^*,” “*E93^2^*,” and “*E93^3^*” have been considered the “type” alleles of the gene, and have been utilized in almost all subsequent studies of *E93* function. These mutations cause an almost complete block in the death of salivary gland cells during metamorphosis. The “E93” mutants eliminate, or reduce, expression of several apoptotic genes in the pupal salivary gland, including *rpr*, *hid*, *crq*, *ark*, and *dronc* (Lee *et al.* 2000), and show low levels of activated Drice (Martin and Baehrecke 2004). In addition, these mutants do not initiate autophagic cell death changes in the salivary glands (Lee and Baehrecke 2001). These phenotypes led to the view that the transcription factor encoded by *E93* is a critical regulator of salivary gland cell death during metamorphosis (Lee *et al.* 2000).

This view of E93 function has been challenged recently by the finding that a separate complementation group, identified by Lee *et al.* (2000) in their lethal screens of the *E93* region, is allelic to *E93*. The alleles of this group (the *E93^4^* through *E93^6^* alleles) are nonsense changes at codons 360, 545, and 783 of the 1165 codon *E93 A* coding sequence (Mou *et al.* 2012). These mutations cause lethality at a much later pupal stage than do alleles of the “*E93^1-3^*” group, producing pharate adults that show numerous defects in structures normally patterned at the pupal stage (Lee *et al.* 2000; Mou *et al.* 2012). Indeed, essentially all cuticular patterning events that normally occur during the pupal stage fail in mutants of this group, suggesting that E93 functions as a key temporal determinant of adult development during metamorphosis (Mou *et al.* 2012). Recent work in other insects strongly supports this conclusion (Ureña *et al.* 2014).

The relationship between the *E93^4-6^* and “*E93^1-3^*” groups of alleles has been puzzling. Alleles of the two groups complement, and, in cell clones alleles of the “*E93^1-3^*” group, show no defects in imaginal patterning, unlike alleles of the *E93^4-6^* group (Mou *et al.* 2012). Complementation of the *E93^4-6^* alleles and “*E93^1^*” has been particularly hard to understand, as the *E93^4-6^* nonsense alleles truncate the coding region at positions upstream of the *E93^1^* nonsense allele. In an attempt to resolve this paradoxical behavior, we localized the “*E93^1-3^*” alleles by deletion mapping and sequencing. Here, we show that the causative mutations in this group are not in fact allelic to *E93*; rather, they are allelic to a nearby gene (*CG6439*) that encodes the β subunit of isocitrate dehydrogenase-3 (Idh3b), a key enzyme of the citric acid cycle. This finding shifts the focus from E93 to Idh3b and/or mitochondrial function in the initiation of salivary gland cell death. We show that Idh3b is required for mitochondrial polarization and the generation of normal ATP levels. For the strongest of the *Idh3b* alleles, embryos from homozygous mutant germline mothers arrest development during early cleavage, consistent with a near-complete block in oxidative phosphorylation. We show that Idh3b is required for mitochondrial fragmentation, an early step in the death program of larval salivary gland cells (Goyal *et al.* 2007), and that, in normal development, tagged Idh3b is expelled from mitochondria during this fragmentation. This observation suggests that Idh3b may be an apoptogenic factor functionally similar to cytochrome *c* and other factors released by mammalian mitochondria in the initiation of cell death. Idh3b is the second TCA cycle enzyme (after malate dehydrogenase-2) shown to be required for the death of larval salivary gland cells (Wang *et al.* 2010), suggesting a key role for the TCA cycle in initiating larval cell death during metamorphosis.

## Materials and Methods

Unless indicated otherwise, all fly stocks used were obtained from the Bloomington *Drosophila* Stock Center (BDSC).

### Generation of E93 region deletions

Defined deletions within the *E93* region were produced by FLP-catalyzed recombination, basically as described by Parks *et al.* (2004). Deletions were generated between the following pairs of insertions (Thibault *et al.* 2004): *P*{*XP*}*Eip93F^d07598^* and *PBac*{*WH*}*Eip93F^f01771^*, *P*{*XP*}*d06373* and *PBac*{*WH*}*Eip93F^f01771^*, and *P*{*XP*}*Eip93F^d07598^* and *PBac*{*WH*}*CG6439^f07670^*. Stocks of each insertion were obtained from the Exelixis Harvard Medical School Collection. Males and females heterozygous for a pair of insertions, carrying an X chromosome marked by *w*, and containing a heat-shock inducible FLP transgene (*hs-FLP122*), were heat-shocked as larvae three times for 30 min each at 37° in plastic vials in a water bath. Emerging adults were then mated to *w*/Y; *TM3*, *Sb*/*TM6B*, *Tb* or *w*/*w*; *TM3*, *Sb*/*TM6B*, *Tb* flies. Deletion recombinants were identified among the progeny by their white eye color, and recombinant chromosomes placed into balanced stock with *TM6B*, *Tb*.

### Germline clones

Germline clones were generated as described by Chou *et al.* (1993). *y w hs-FLP122*/*w*; *FRT82B Idh3b*/*FRT82B P*{*ovo^D1^*}*^18^* female larvae were heat-shocked at 37° three times during larval development. Control females not subject to heat shock produced no eggs, confirming the presence and efficacy of *P*{*ovo^D1^*}*^18^*. However, heat-shocked females containing *Idh3b*-homozygous clones in their germlines produced numerous, normal-appearing eggs. A major concern in these experiments was the elimination of extraneous lethals from the *Idh3b* mutant chromosomes. Each of the three *Idh3b* alleles was subject to multiple rounds of recombination to generate chromosomes for each that survive to pupariation when homozygous. These chromosomes were then recombined with a homozygous viable *FRT82B* chromosome to generate *FRT82B Idh3b* chromosomes. All recombinant chromosomes used for clone production survive to pupariation when homozygous.

### Amplification and sequencing of mutants

For each of the “*E93^1-3^*” alleles, the region between the *PBac*{*WH*}*Eip93F^f01771^* and *PBac*{*WH*}*CG6439^f07670^* insertions was amplified and cloned as three ∼3 kb subfragments into pJET 2.1, which were then sequenced with primers spaced at 800 bp intervals (primer sequences available upon request). A single nucleotide difference within the *Idh3b* gene was found for each mutation.

### Fly transformation

All transgenes were generated by standard molecular methods. DNA was prepared with PureYield Midi preps (Promega), and resuspended in injection buffer (5 mM potassium chloride, 0.1 mM sodium phosphate, pH 7.8) at a concentration of 1 µg/µl. Staged embryos of appropriate genotype were dechorionated in bleach, and injected under oil with pulled glass capillary needles using a micromanipulator (Narishige) controlled with a N_2_ driven Picospritzer (General Valve Corporation). All constructs were injected into one or more of the following *ϕc31* integrase and *attP* site strains: *y^1^ M*{*vas-int.DM*}*ZH-2A w*; *M*{*3XP3-RFP.attP′*}*ZH-22A* (BDSC line 24481), *y^1^ M*{*vas-int.DM*}*ZH-2A w*; *M*{*3XP3-RFP.attP′*}*ZH-68E* (BDSC line 24485), and/or *y^1^ M*{*vas-int.DM*}*ZH-2A w*; *M*{*3XP3-RFP.attP′*}*ZH-51D* (BDSC line 24483).

### Transgene constructions

The *Idh3b* rescue construct was derived by amplifying the *Idh3b* region using wild-type genomic DNA as template, followed by cloning as a *Not*I–Acc65I fragment into *pUASTattB* (Bischof *et al.* 2007). The genomic fragment tested includes the entire Idh3b A form transcribed region, as well as 198 bp of upstream sequence. The HA/FLAG tagged Idh3b construct was ordered directly from the *Drosophila* Genome Resource Center Tagged ORF collection (accession number UFO02744).

### ATP assays

Assays were performed with the Enlighten ATP Bioluminescence Kit (Promega) according to the manufacturer’s protocols. Briefly, pupae aged 18 hr after puparium formation (APF) were collected, and ground in 100 µl of homogenization buffer (five animals per sample); 20 µl was removed for Bradford Protein Assays (Sigma), and the rest of each sample was boiled 5 min and spun 3 min at 14,000 rpm to remove debris. The supernatant was diluted 1:1000× with ATP-free water, and 10 µl aliquots were mixed with 100 µl rL/L reagent in white 96-well plates, and read immediately on a Varioskan Luminometer (Thermo Scientific), along with a freshly prepared ATP standard series. Three replicates were assayed for each sample, and three readings were conducted for each experiment. ATP concentration was determined by comparison to the ATP standard curve, and normalized to total protein.

### Antibody/mitotracker/lysotracker staining

For MitoTracker and LysoTracker staining, tissues were dissected at room temperature in Phosphate Buffered Saline (PBS), rinsed briefly in PBS, and incubated for 5 min in 100 nM MitoTracker RedCMXRos, 100 nM MitoTracker Deep Red, or 50 nM LysoTracker Red (all reagents from Life Technologies). The samples were quickly rinsed three times in PBS, and fixed in 4% Paraformaldehyde in PBS for 1 min at room temperature. The samples were then rinsed in PBS with 0.05% Triton X (PBSTx), stained for 5 min with 2 µg/ml DAPI, rinsed twice with PBSTx, and mounted in 50% glycerol/50% PBS. Antibody staining was performed as described previously (Kankel *et al.* 2004). Antibodies used in this study were monoclonal mouse anti-Engrailed (cell line gift from N. Patel, Univ. California, Berkeley), monoclonal rat anti FLAG (BioLegend), Cy3 Donkey anti mouse, and Cy3 Donkey anti rat (Jackson Immunolabs). Images were collected with a Nikon A1 confocal microscope and a Zeiss SV11 fluorescent stereomicroscope with GFP filters.

### Data availability

All *Drosophila* strains used or generated in this study are available on request. The authors state that all data necessary for confirming the conclusions presented in the article are represented fully within the article.

## Results

**Figure 1:**
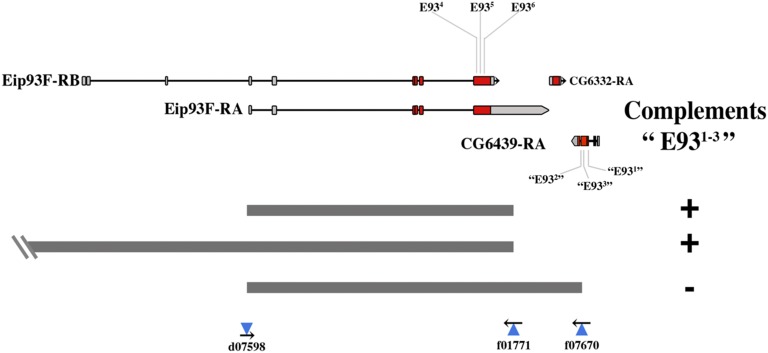
Deletion mapping of the “*E93^1-3^*” alleles. At the top are shown the transcripts of the *E93*, *CG6332*, and *CG6439* genes, and at the bottom are shown the extents of deletions generated by recombination between FRT elements carried by the indicated insertions. At the right, the ability of each deletion to complement the “*E931-3*” alleles is indicated. Coding regions of each transcript are indicated in red.

**Figure 2:**
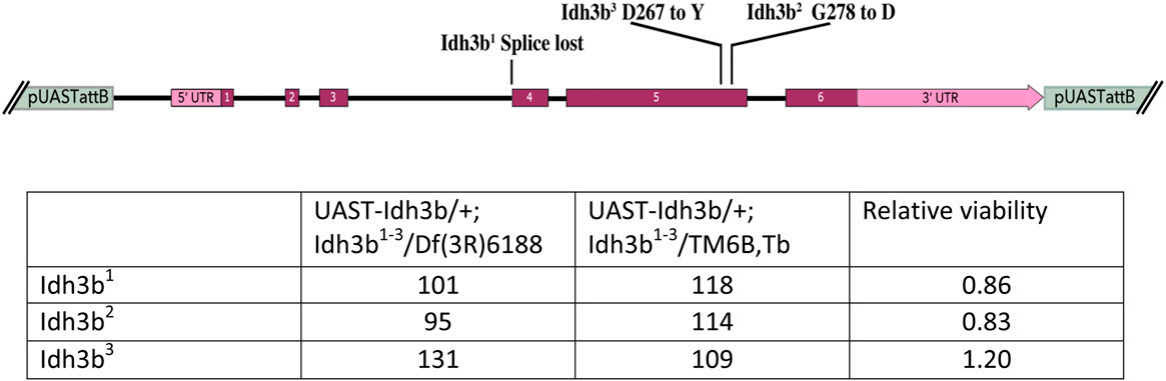
Rescue of the *Idh3b* mutants by an *Idh3b*^+^ transgene. (A) The *Idh3b* A form transcribed region, showing the locations of exons, coding sequences, untranslated regions, the *Idh3b* alleles, and the extent of the *Idh3b* rescue fragment. The indicated fragment was cloned into *pUASTattB* and transformants generated. These transformants rescue the *Idh3b* mutants in the absence of a Gal4 driver. (B) Ability of the *Idh3b* rescue construct to rescue the lethality of *Idh3b* mutant hemizygotes. Crosses to test rescue were of the form *w*; *UAST-Idh3b*/*SM6*; *Idh3b^1-3^*/*TM6B*, *Tb* males x *w*/*w*; *Df*(*3R*)*Exel6188*/*TM6B* females. Adult progeny numbers are shown only for *Idh3b^1^*^-^*^3^*/*Df*(*3R*)*Exel6188* and *Idh3b^1^*^-^*^3^*/*TM6B*, *Tb* progeny that carry the *UAST-Idh3b* transgene. Almost complete rescue of survival to adulthood is seen for all three alleles. For the *Idh3b^2^* mutant, rescue of salivary gland death was also examined. Of nine *UAST-Idh3b*/+; *Idh3b^2^*/*Df*(*3R*)*Exel6188* pupae dissected at 24–30 hr after puparium formation, none contained salivary glands.

To explore the relationship between the *E93^4-6^* and “*E93^1-3^*” complementation groups, our first goal was to generate a true null allele. This was accomplished by FLP-mediated recombination between FRT elements (Parks *et al.* 2004) carried by existing transposon insertions that lie at either end of the major coding region of the gene [insertions *d07598* and *f01771* (Thibault *et al.* 2004)] (Figure 1). The deletion generated, *E93*^Δ^*^1^*, lacks the entire E93A coding region, and all but the 5′-most 32 codons of the E93B isoform coding sequence. Homozygotes for *E93*^Δ^*^1^* survive to the pharate adult stage, and show cuticular defects essentially identical to those shown by homozygotes for alleles of the *E93^4-6^* group. As expected, *E93*^Δ^*^1^* fails to complement alleles of this group. However, *E93*^Δ^*^1^* fully complements the three alleles of the “*E93^1-3^*” group. This result strongly suggests that alleles of the latter group affect some product other than E93. To identify this product, we generated two additional deletions: one includes the *E93*^Δ^*^1^* deletion and extends to the left (produced by FLP catalyzed recombination between the insertions *d06373* and *f01771*), while the other includes *E93*^Δ^*^1^* and extends to the right (produced by recombination between the insertions *d07508* and *f07670*) (Figure 1). The former deletion complements the “*E93^1-3^*” group, whereas the latter does not, indicating that the “*E93^1-3^*” alleles lie close to the right of the *E93* coding sequence. Amplification and sequencing of this region from each of the mutant alleles revealed that all three carry mutations within a nearby gene (CG6439), which encodes a 370 amino acid protein orthologous to the β subunit of human isocitrate dehydrogenase 3. “*E93^1^*” is an A to T change in the AG splice acceptor at the 5′ boundary of exon 4 of this gene; “*E93^2^*” is a G to A missense allele causing the change of G278 to D; and “*E93^3^*” is a G to A missense allele causing the change of D267 to Y (Figure 2). Allelism of the *E93^1-3^* mutations with *CG6439* was confirmed by essentially complete rescue of their lethality by a 3.0 kb genomic fragment containing the *CG6439* gene (see Figure 2 for details of the construct used, and data on its rescue ability). Henceforth, these alleles will be called *Idh3b^1^*, *Idh3b^2^*, and *Idh3b^3^*, respectively. The full complementation of *E93*^Δ^*^1^* and “*E93^1^*” indicates that the *E93* C-terminal nonsense mutation present in the latter is incidental, and does not compromise any essential function of *E93*.

A second reason that the *Idh3b* alleles were thought to be allelic to *E93* is that *E93* transcripts were reported to be absent in *Idh3b^3^* mutants, and strongly reduced in *Idh3b^2^* mutants (Lee *et al.* 2000). However, we find that antibody staining for E93 protein is normal in salivary glands from heterozygotes for *Idh3b^3^* and a deficiency that removes the *Idh3b* and *E93* genes [*Df*(*3R*)*Exel6188*] (Supplemental Material, Figure S1). E93 staining is also seen *Idh3b^2^*/*Df*(*3R*)*Exel6188* salivary glands, although the level of staining is reduced compared to wild type. This reduction is likely due to developmental arrest, as E93 staining is normal in salivary glands from *Idh3b^2^*/*E93*^Δ^*^1^* heterozygotes. These observations indicate that the *Idh3b* mutants are not transcript null alleles of *E93*.

**Figure 3:**
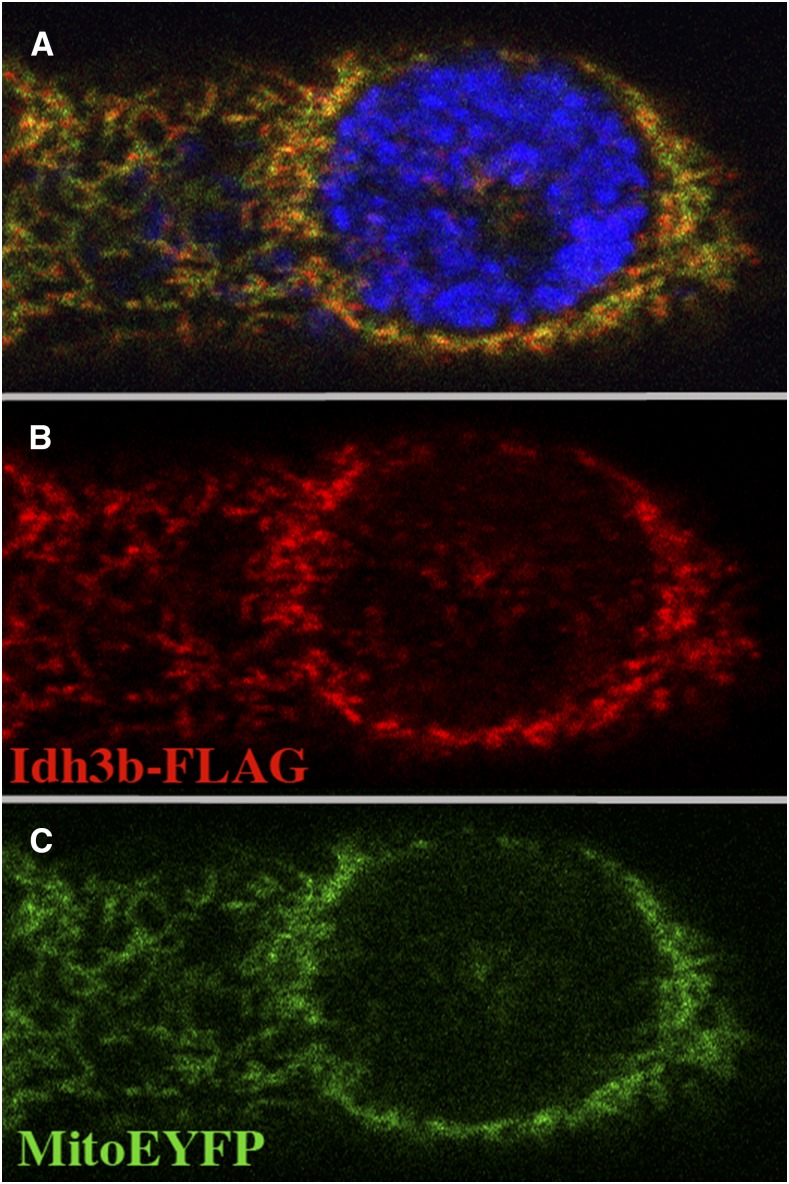
FLAG-tagged Idh3b localizes to mitochondria. A tracheal cell from a mid third-instar larva of the genotype *UAS-HA*/*FLAG-Idh3b*/+; *tub-Gal4*/*mito-EYFP* that has been stained with anti-FLAG (red) and the DNA dye DAPI (blue) is shown. FLAG-Idh3b (red) (B) and mito-EYFP (green) (C) colocalize (see merged image in A).

Three forms of isocitrate dehydrogenase are present in humans: IDH1 and IDH2 are NADP-dependent enzymes, whereas IDH3 is NAD dependent. IDH1 is found within the cytosol and in peroxisomes, while IDH2 and IDH3 localize to mitochondria. IDH2 is known to function in the elimination of reactive oxygen species generated by electron transport (Jo *et al.* 2001), while IDH3 is thought to function within the tricarboxylic acid (TCA) cycle to catalyzes the oxidative decarboxylation of isocitrate to α-ketoglutarate. IDH3 is a heterotetramer, consisting of two α subunits, one β subunit, and one γ subunit. *Drosophila CG6439* is most similar in sequence to the β subunit of IDH3 (61% identity and 77% similarity), with the next highest similarity being to the IDH3γ subunit (51% identity and 68% similarity). To localize *Drosophila* Idh3b, we drove expression of FLAG-tagged Idh3b (*Drosophila* Genome Resource Center) using the tubulin-Gal4 driver, and compared its localization to that of mito-EYFP, which localizes to mitochondria (LaJeunesse *et al.* 2004). Tagged Idh3b and mito-EYFP colocalize in all cell types examined (imaginal disc, tracheal, and salivary gland cells) (Figure 3), consistent with the presence of a predicted mitochondrial targeting peptide at the N-terminus of the protein (TargetP prediction; Emanuelsson *et al.* 2000), and the identification of Idh3b within the proteome of the *Drosophila* mitochondrial matrix (Chen *et al.* 2015).

**Figure 4:**
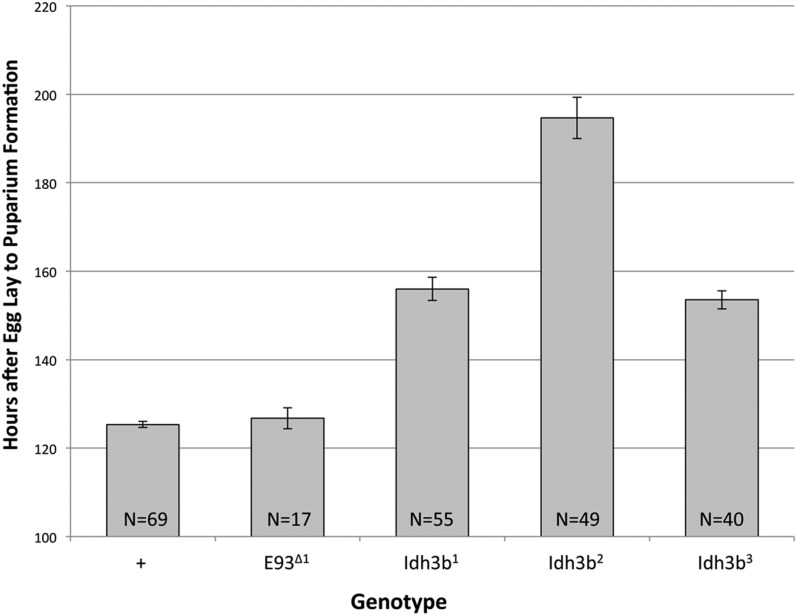
The *Idh3b* alleles cause slowed development. Average egg to puparium development times are indicated for heterozygotes of +, *E93*^Δ^*^1^*, and each of the *Idh3b* alleles with *Df*(*3R*)*Exel6188*. The *Idh3b* alleles cause increases of 24–55% in egg to puparium development time, whereas the *E93*^Δ^*^1^* allele has no effect. *N* = total number of puparia scored.

As would be expected for impairment of mitochondrial function, *Idh3b* mutants develop more slowly than wild type. Egg to puparium development time was determined for each *Idh3b* allele when heterozygous with *Df*(*3R*)*Exel6188*. Compared to wild-type hemizygotes, *Idh3b^1^*, *Idh3b^2^*, and *Idh3b^3^* hemizygotes show increases in average egg to puparium development of 24, 55, and 22%, respectively (Figure 4). Despite this developmental delay, the egg to puparium survival of the *Idh3b* mutant hemizygotes (∼80%) does not differ significantly from wild-type control hemizygotes. For all three mutant alleles, anterior spiracle eversion is often abnormal at pupariation, and, for *Idh3b^2^* mutants, many puparia resemble elongate sclerotized larvae. Head eversion, which marks the transition between the prepupal and pupal periods, occurs in about half of *Idh3b^1^* and *Idh3b^2^* hemizygotes (22/39 and 19/38 animals, respectively), but in essentially all *Idh3b^3^* hemizygotes (37/38 animals). Based on developmental delay, morphological abnormalities at pupariation, and head eversion failure, the *Idh3b* alleles can be ordered in severity as *Idh3b^2^* > *Idh3b^1^* > *Idh3b^3^*.

**Figure 5:**
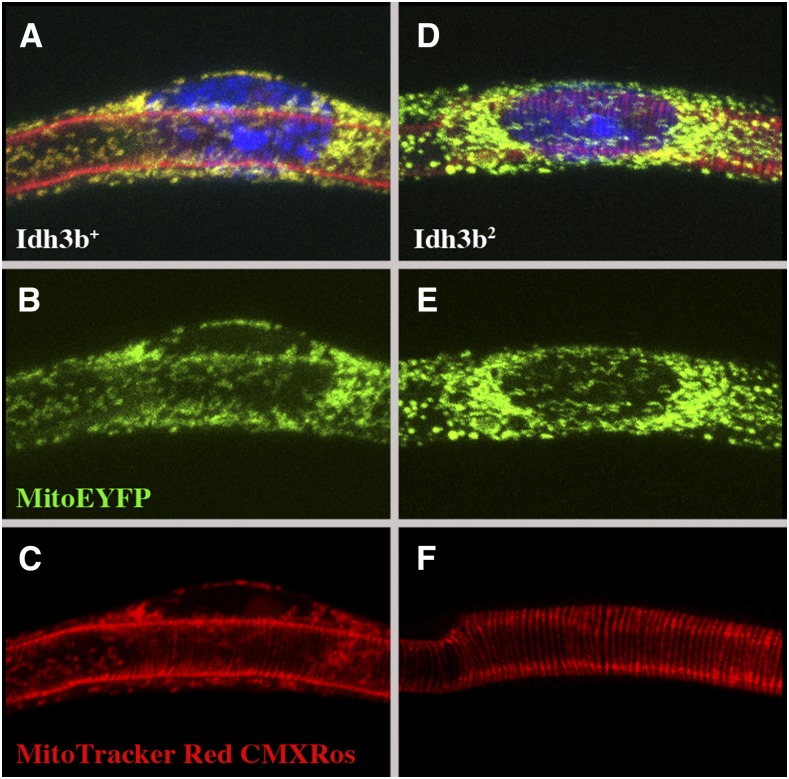
Loss of mitochondrial polarization in *Idh3b^2^* mutants. (A–C) a tracheal cell from a wandering third instar *mito-EYFP Idh3b^+^/Df*(*3R*)*Exel6188* larva stained with the dye MitoTracker Red CMXRos, which is specific for polarized (functional) mitochondria. MitoTracker staining (C) is coincident with mito-EYFP (B) (see merged image in A). (D–F) a tracheal cell from a wandering third instar *mito-EYFP Idh3b^2^*/*Df*(*3R*)*Exel6188* larva. MitoTracker Red CMXRos staining (F) is absent from mitochondria (labeled in E by mito-EYFP; merged image in D). The striated tubes showing red fluorescence in A, C, D and F are the tracheae; this fluorescence results from the combined effects of cuticle autofluorescence and affinity of the dye for tracheal cuticle. In A and D, DNA is stained by DAPI (blue).

**Figure 6:**
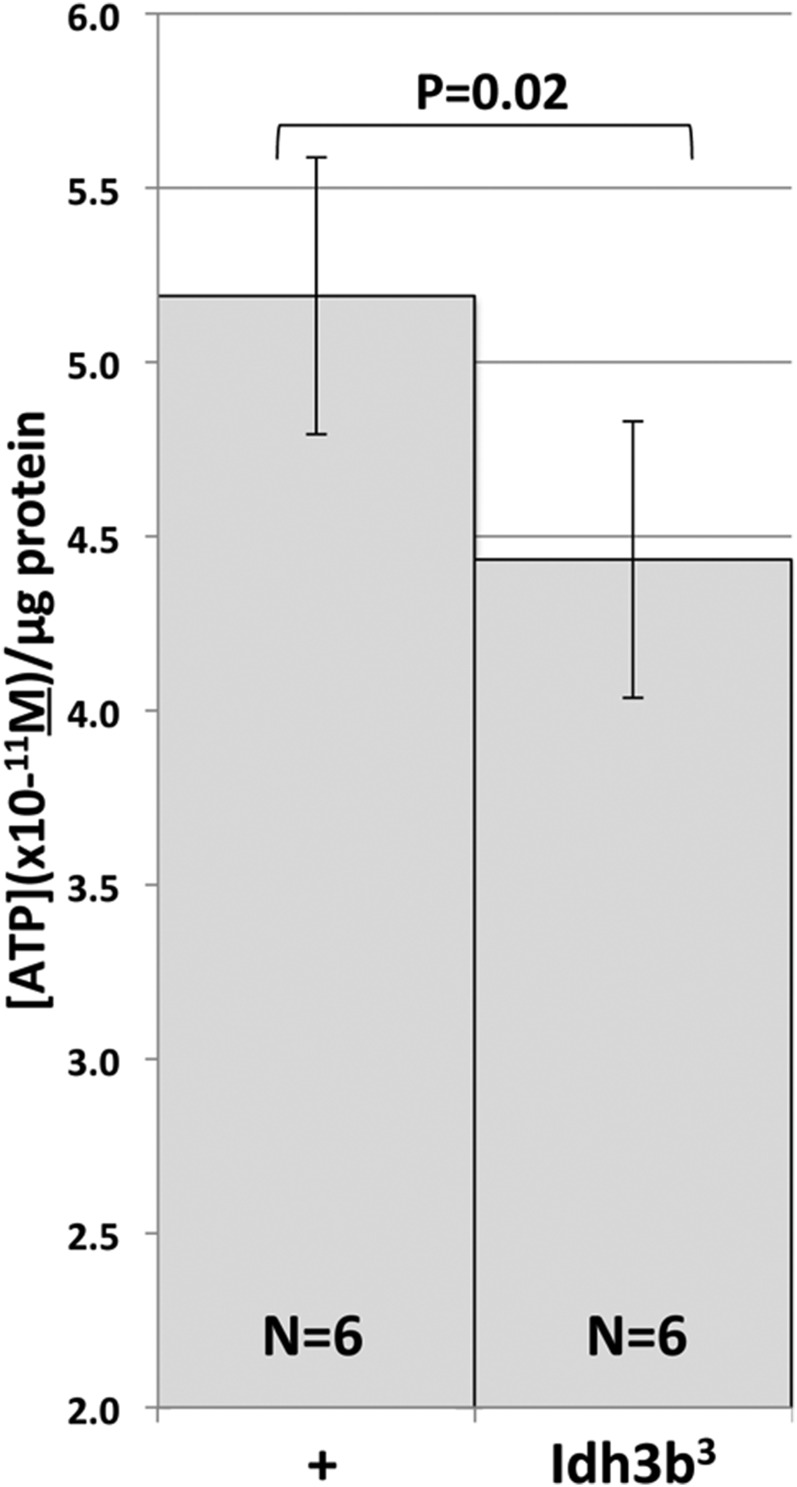
ATP levels are reduced in *Idh3b^3^* mutants. ATP levels are shown for +/*Df*(*3R*)*Exel6188* and *Idh3b^3^*/*Df*(*3R*)*Exel6188* 18 hr pupae. The *Idh3b^3^* mutant shows a decline in ATP level of ∼15% relative to wild type (*P* = 0.02). Six independent samples were tested for each genotype.

Disruption of the TCA cycle in the *Idh3b* mutants would be expected to result in mitochondrial dysfunction. To test this possibility, we stained living cells with the dye MitoTracker Red CMXRos (ThermoFisher), which is taken up only by polarized mitochondria. Tracheal cells from late third-instar larvae were examined, as these cells have prominent mitochondria, and are readily accessible to the MitoTracker dye. In *Idh3b*^+^ *mito-EYFP*/*Df*(*3R*)*Exel6188* control animals, essentially all mitochondria in these cells stain with the MitoTracker dye, whereas in *Idh3b^2^ mito-EYFP*/*Df*(*3R*)*Exel6188* cells, MitoTracker staining is absent (Figure 5). MitoTracker staining is also reduced, although to a lesser extent, in *Idh3b^1^ mito-EYFP*/*Df*(*3R*)*Exel6188* and *Idh3b^3^ mito-EYFP*/*Df*(*3R*)*Exel6188* cells (not shown). The dye MitoTracker Deep Red, which is largely, but not completely, dependent on mitochondrial polarization, gave similar results (not shown). To test whether this mitochondrial dysfunction is associated with reduced ATP production, we compared ATP levels in *Idh3b* mutant and wild-type animals at 18 hr APF, a time at which oxidative phosphorylation, rather than glycolysis, has become an important source of ATP (Wang *et al.* 2010; Tennessen *et al.* 2011, 2014). The weakest allele (*Idh3b^3^*) was selected for examination because, unlike *Idh3b^1^* and *Idh3b^2^*, *Idh3b^3^*-mutant animals do not arrest development until well after 18 hr APF, allowing accurate staging in the prepupal/early pupal periods. We find that ATP levels in *Idh3b^3^*/*Df*(*3R*)*Exel6188* animals are ∼15% lower (*P* = 2%) than in +/*Df*(*3R*)*Exel6188* controls at 18 hr APF (Figure 6). Since *Idh3b^3^* is the weakest of the alleles, this reduction in ATP is a minimal estimate of the requirement for Idh3b.

In previous work, the *Idh3b* mutations (then thought to be *E93* alleles) were found to cause strong persistence of larval salivary glands in the pupa (Lee *et al.* 2000). We have confirmed this observation. Loss of glands was monitored in intact pupae in which GFP expression was driven specifically within salivary glands by the *Sg-Gal4* driver (Wang *et al.* 2008). In control +/*Df*(*3R*)*Exel6188* pupae, salivary gland loss is complete at between 21 and 23 hr APF at room temperature (Figure S2 and Figure S3). However, salivary glands persist beyond 30 hr APF in the three *Idh3b* mutant hemizygotes. In contrast, *E93*^Δ^*^1^* hemizygotes cause only slightly increased persistence of salivary glands relative to wild type (Figure S2 and Figure S3). The nonsense alleles *E93^4^*, *E93^5^*, and *E93^6^* behave similarly. *E93*^Δ^*^1^* hemizygotes also show no developmental delay during larval development (Figure 4), unlike the *Idh3b* mutants.

**Figure 7:**
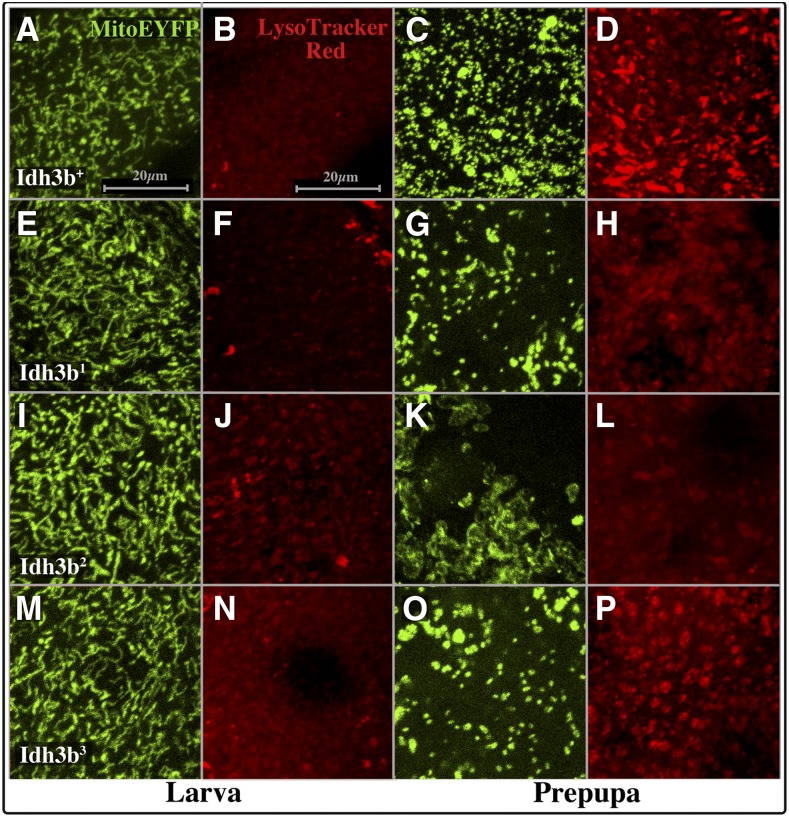
Idh3b is required for mitochondrial fragmentation and increase in acidic compartments after pupariation in salivary gland cells. Panels show salivary gland cells of the genotypes *mito-EYFP Idh3b^+^*/*Df*(*3R*)*Exel6188* (A–D), *mito-EYFP Idh3b^1^*/*Df*(*3R*)*Exel6188* (E–H), *mito-EYFP Idh3b^2^*/*Df*(*3R*)*Exel6188* (I–L), and *mito-EYFP Idh3b^3^*/*Df*(*3R*)*Exel6188* (M–P). In mid third-instar larvae, mitochondria are unfragmented (A, E, I, and M) and LysoTracker staining is very weak (B, F, J, and N) in all genotypes shown. However, in 8–12 hr APF prepupal glands (C, D, G, H, K, L, O, and P), mitochondria have undergone extensive fragmentation in *mito-EYFP Idh3b^+^*/*Df*(*3R*)*Exel6188* animals (C), and LysoTracker staining has increased dramatically (D), consistent with the initiation of autophagic cell death. In glands from *mito-EYFP Idh3b^1^*/*Df*(*3R*)*Exel6188* and *mito-EYFP Idh3b^3^*/*Df*(*3R*)*Exel6188* prepupae, mitochondrial fragmentation is not as extensive (G and O), and LysoTracker staining increases to a lesser extent (H and P). In glands from *mito-EYFP Idh3b^2^*/*Df*(*3R*)*Exel6188* prepupae, mitochondria do not fragment, but appear clumped (K), and LysoTracker staining fails to increase (L). All panels are at the same magnification (see scale bars in A and B).

**Figure 8:**
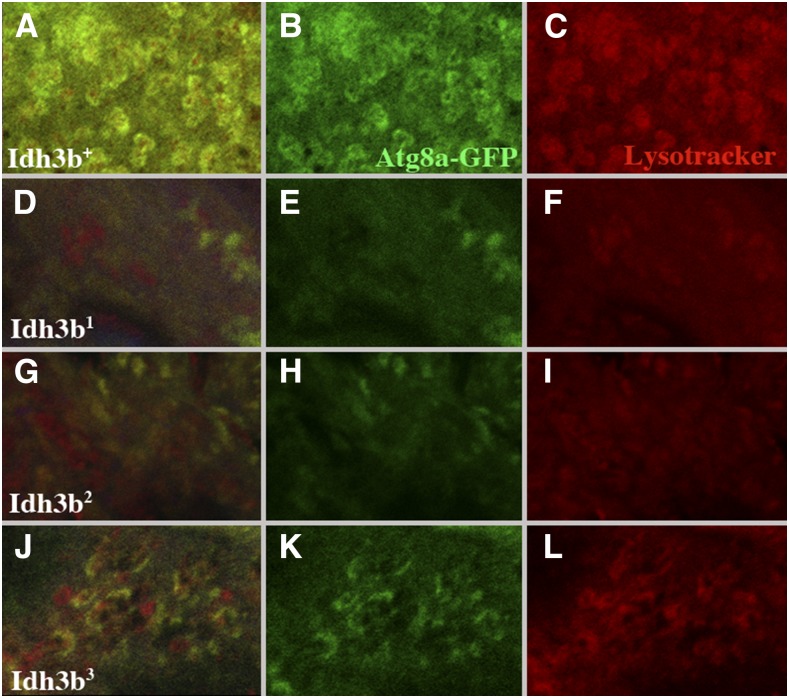
Autophagy is blocked in *Idh3b*-mutant salivary gland cells. All images show salivary gland cells from 8 to 12 APF prepupae, in which expression of *UAS-Atg8a-GFP* is driven by *Sg-Gal4*. Atg8a-GFP is shown in green, and LysoTracker staining is in red. (A–C) In *Idh3b*^+^/*Df*(*3R*)*Exel6188* cells, Atg8a-GFP labeled vesicles are densely packed, and these vesicles colabel with LysoTracker, identifying them as autolysosomes. (D–I) In *Idh3b^1^*/*Df*(*3R*)*Exel6188* and *Idh3b^2^*/*Df*(*3R*)*Exel6188* cells, Atg8a-GFP-labeled vesicles are dramatically reduced in number, and LysoTracker staining is much reduced, indicating an almost complete block in autophagy. (J–L) *Idh3b^3^*/*Df*(*3R*)*Exel6188* cells show an intermediate phenotype, in which Atg8a-GFP vesicles are partially formed, and LysoTracker staining is weakened, and incompletely associated with Atg8a-GFP fluorescence.

The death of larval salivary gland cells is known to involve autophagy (Lee and Baehrecke 2001). To monitor autophagy we used the dye LysoTracker (Thermo Fisher), which labels acidic cellular compartments, including lysosomes and autolysosomes, and Atg8a-GFP, which labels the membranes of autophagosomes and autolysosomes (Scott *et al.* 2004). In wild type, LysoTracker staining in salivary gland cells increases dramatically in the prepupal stage (Figure 7, B and D). When Atg8a-GFP is driven by *Sg-Gal4*, very little fluorescence is seen at 0 hr APF, but, by 13 hr APF salivary gland cytoplasm contains densely packed GFP-labeled vesicles (Figure 8, A–C). These vesicles colabel with LysoTracker, identifying them as autolysosomes. The high density of these autolysosomes accounts for the rapid degradation of the salivary gland cytoplasm at this stage in wild type. In *Idh3b*^1^ and *Idh3b*^2^ mutants, LysoTracker staining increases only slightly, or not at all, after pupariation, and the number of Atg8a-GFP-labeled vesicles at 13 hr APF is dramatically reduced (Figure 7, H and L and Figure 8, D–I). *Idh3b^3^* mutants have an intermediate phenotype: LysoTracker increases after pupariation, but less so than wild type (Figure 7P), and at 13 hr APF many partially formed Atg8a-GFP-labeled vesicles are present (Figure 8, J and K) These partially formed vesicles are associated with LysoTracker staining, but the association is not as complete as in wild type. Taken together, these observations are consistent with cytological observations of Lee and Baehrecke (2001) that demonstrate a failure to initiate autophagy in *Idh3b* mutant salivary glands.

**Figure 9:**
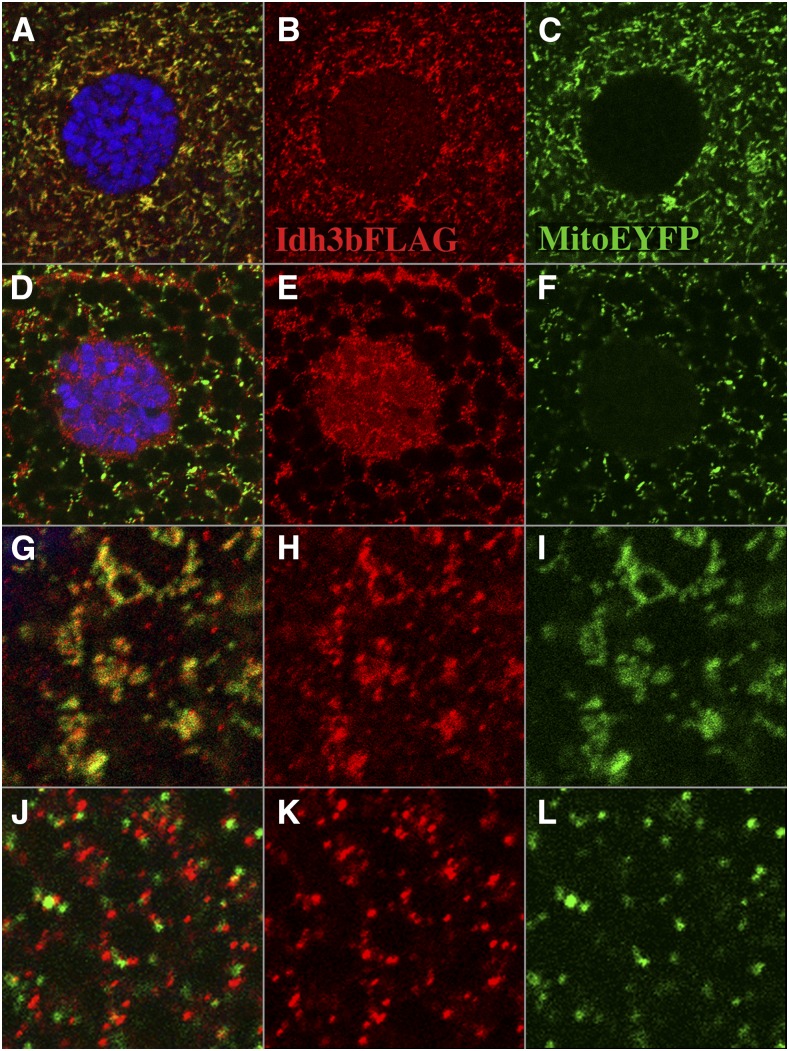
Expulsion of Idh3b from mitochondria after fragmentation. All images are from larvae of the genotype *UAS-HA/FLAG-Idh3b/+*; *tub-Gal4*/*mito-EYFP*. (A–C) Salivary gland cell from a mid third-instar larva. FLAG-Idh3b and mito-EYFP colocalize. The nucleus (marked by DAPI staining in blue) shows no mito-EYFP fluorescence, and is very weakly labeled for FLAG-Idh3b. (D–F) Salivary gland cell from a late (wandering) third-instar larva. Mitochondrial fragmentation has taken place. FLAG-Idh3b staining and mito-EYFP are no longer coincident (see merged image in D). Note that FLAG-Idh3b staining within the nucleus has become more intense (E), but that mito-EYFP staining continues to be excluded from the nucleus (F). The dark lacunae in these images are secretory vacuoles, which are prominent at this time. (G–I) High magnification of part of a mid third-instar salivary gland cell. Note that FLAG-Idh3b staining (H) and mito-EYFP (I) largely colocalize (G). (J–L) High magnification of part of a late third-instar salivary gland cell. FLAG-Idh3b puncta (K) and mito-EYFP (L) are now almost mutually exclusive (merged image in J).

To explore further the role of Idh3b in salivary gland elimination, we examined the behavior of mitochondria in mutant larval salivary glands. In wild type, salivary gland mitochondria undergo extensive fragmentation in late larvae (Goyal *et al.* 2007) (Figure 7, A–D). This fragmentation is thought to be an early initiating step in the death of the glands. We find that mitochondrial fragmentation is absent or very much reduced in *Idh3b^2^* mutants compared to wild type (Figure 7, I–L). Mutant mitochondria remain elongated, and appear clumped together. Weaker reduction in fragmentation is seen in *Idh3b^1^* (Figure 7, E–H) and *Idh3b^3^* (Figure 7, M–P) mutants. Surprisingly, mitochondrial localization of Idh3b is stage-specific in salivary gland cells. In glands from *Idh3b*^+^ mid third-instar larvae, FLAG-Idh3b, and mito-EYFP colocalize (Figure 9, A–C and G–I). However, in prepupae, after mitochondrial fragmentation, FLAG-Idh3b and mito-EYFP fluorescence show near mutually exclusive punctate localization (Figure 9, D–F and J–L). Strikingly, after mitochondrial fragmentation, FLAG-Idh3b puncta are present within the nucleus, while mitochondria (*i.e.*, mito-EYFP) are not (Figure 9, D–F). Other mitochondrial proteins, including the TCA cycle enzyme fumarase, are known to become localized to the nucleus under certain conditions [see Tang (2015) for review]. Whether the nuclear localization of Idh3b seen here after mitochondrial fragmentation is of functional significance is not clear.

**Figure 10:**
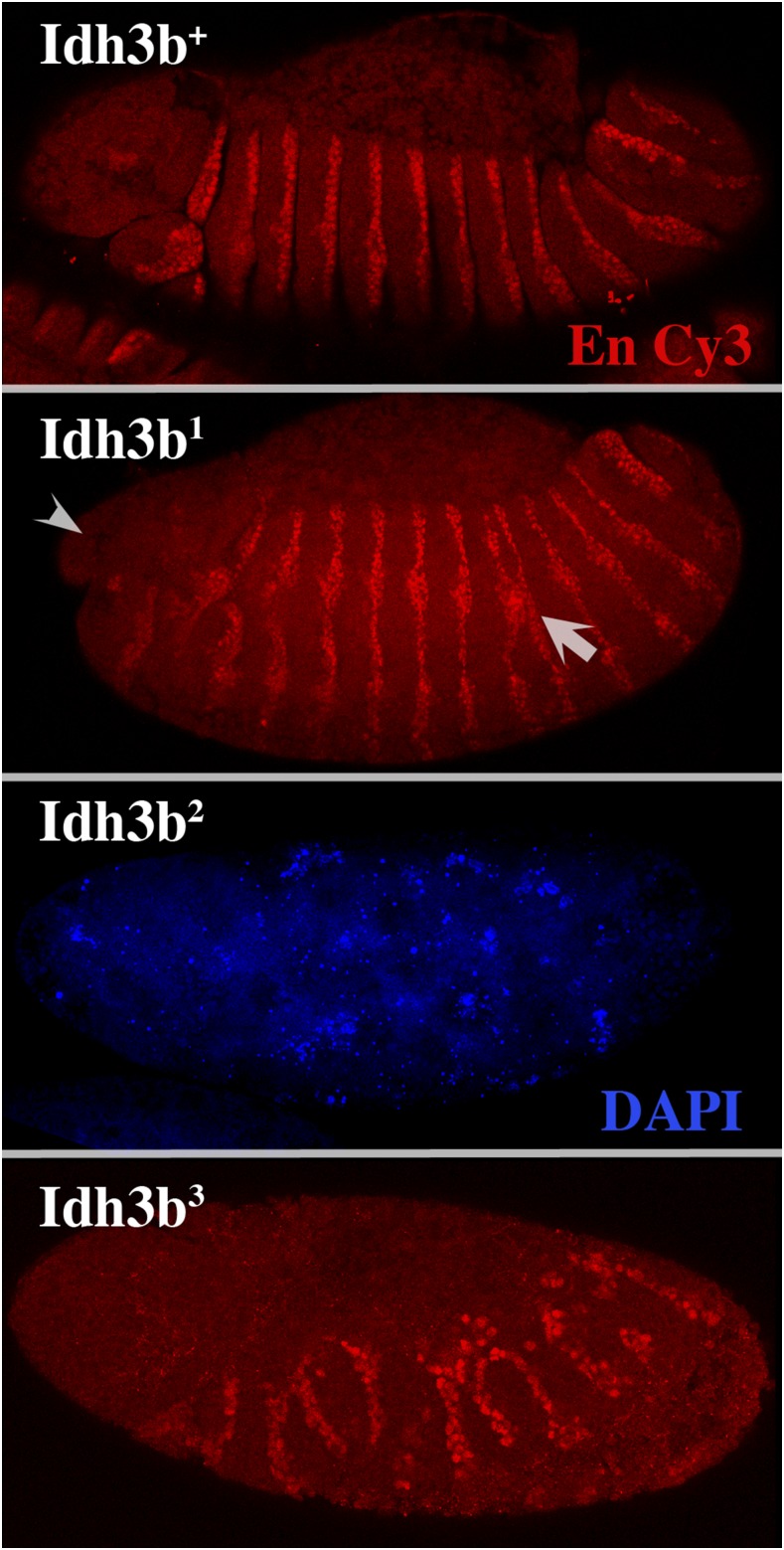
Absence of maternally supplied Idh3b causes developmental arrest. Embryos shown are from mothers whose germlines were homozygous for the indicated *Idh3b* alleles crossed to *Df*(*3R*)*Exel6188*/*TM6B* fathers. For embryos from *Idh3b*^+^, *Idh3b^1^*, and *Idh3b^3^* homozygous germline mothers, the segmentation protein engrailed (En) is stained using Cy3-labeled anti-engrailed (red). Since embryos from *Idh3b^2^* mutant germline mothers all arrest prior to segmentation, the *Idh3b^2^* mutant embryo shown is stained instead with DAPI (blue), which stains nuclei. Most embryos from *Idh3b^1^* germline mothers segment, but engrailed stripes usually show spacing defects or are fused with one another (arrow). The head region (arrowhead) is almost always reduced. Embryos from *Idh3b^2^* germline mothers all arrest during cleavage, and contain nuclei of variable size. Embryos from *Idh3b^3^* germline mothers arrest at stages ranging from cleavage to cuticle formation; almost all of those that express engrailed have grossly abnormal striping patterns, like the embryo shown here.

To test whether maternally contributed Idh3b is important for normal development, we used the *ovo^D^* dominant female sterile method (Chou *et al.* 1993) to generate mothers whose germlines were homozygous mutant for each of the *Idh3b* alleles. These females were crossed to males heterozygous for *Df*(*3R*)*Exel6188*, which is deficient for *Idh3b*. Remarkably, females whose germlines are homozygous for the *Idh3b* alleles produce large numbers of normal-appearing eggs, indicating that the female germline does not require autonomous Idh3b function. However, the embryos that develop from these eggs are highly abnormal. For *Idh3b^2^*-mutant germline mothers, all embryos arrest early during cleavage (Figure 10). Nuclei in arrested embryos are highly variable in size, and often appear incompletely separated, suggesting extensive mitotic abnormalities. These defects are similar to those caused by anoxia at this stage (DiGregorio *et al.* 2001; Foe and Alberts 1985), consistent with an almost complete block in oxidative phosphorylation. Almost all embryos from *Idh3b^3^*-mutant germline mothers also fail to complete embryogenesis. However, these embryos fare much better, with the majority surviving to the blastoderm stage or beyond. About half show some degree of segmentation, as indicated by the development of engrailed-expressing stripes. For most such embryos, these stripes are highly abnormal, being fragmented and showing spacing irregularities (Figure 10), and the head region is strongly reduced. Surprisingly, the *Idh3b^1^* allele has much weaker effects than *Idh3b^3^* in maternal germline clones. This is despite *Idh3b^1^* being significantly stronger than *Idh3b^3^* in its effects on homozygotes from heterozygous mothers. Almost all embryos from *Idh3b^1^* mutant germline mothers survive to segmented stages, although segmentation defects are frequent (Figure 10); ∼15% of such embryos hatch, with a few surviving to pupariation and adulthood.

## Discussion

We report here that the three mutations initially described as the “type alleles” of *E93* are in fact alleles of *Idh3b*, which encodes the β subunit of NAD-dependent isocitrate dehydrogenase—a key mitochondrial enzyme of the TCA cycle. This finding is important because almost all studies aimed at understanding E93 function have relied upon these mutations. Reports of the interaction of *E93* with other genes involved in the ecdysone response hierarchy, as well as the regulation by *E93* of genes involved in cell death and autophagy, will all have to be revisited. Most importantly, the view that E93 serves as a dedicated regulator of cell death will need to be revised. We generate an allele of *E93* (*E93*^Δ^*^1^*) that is deleted for almost the entire *E93* coding sequence. This allele behaves essentially the same as the *E93^4-6^* alleles described previously (Mou *et al.* 2012), consistent with E93 functioning as a temporal identity determinant for pupal development (Mou *et al.* 2012; Ureña *et al.* 2014).

The three *Idh3b* mutations were misidentified as *E93* alleles primarily because one of them (*Idh3b^1^*, called initially *E93^1^*) was found to be associated with a nonsense change near the 3′ end of the *E93* coding sequence (at codon 995 of the E93 A isoform). Allelism with *E93* seemed to make good sense, as the three alleles had the expected phenotype (death after pupariation). However, we find that hemizygotes for *Idh3b^1^* are rescued to adulthood by an *Idh3b^+^* transgene, indicating that the nonsense change in *E93* in this mutant is not responsible for its phenotypic effects. Indeed, this nonsense change has no apparent phenotypic effect, as *E93*^Δ^*^1^*/*Idh3b^1^* heterozygotes are fully viable and appear wild type as adults. The *E93* nonsense change associated with *Idh3b^1^* is almost certainly an incidental EMS-induced change. At the doses of EMS used in the screen that produced the *Idh3b* alleles (25 and 37 mM) (Lee *et al.* 2000), the expected frequency of base changes induced is about one altered base pair in 300–400 kb (Blumenstiel *et al.* 2009; Cooper *et al.* 2008). Therefore, the expected frequency of such incidental changes in the *E93* coding sequence (∼3300 bp) is ∼1% of treated chromosomes.

In their initial description of the “*E93^1-3^*” alleles, Lee *et al.* (2000) performed a “rescue” experiment, in which they showed that driving expression of E93 in salivary glands at the larval stage by use of *forkhead*-Gal4 causes elimination of the glands in an “*E93^1^*” (*i.e.*, *Idh3b^1^*) mutant background. This result was taken as supporting evidence that “*E93^1-3^*” are alleles of *E93*, and that E93 plays a key role in directing larval cell death. However, a significant weakness of this experiment is that E93 expression was driven in salivary glands at the larval stage, rather than at the pupal stage, when E93 expression and salivary gland death normally occur. In imaginal discs, ectopic expression of a wide variety of cell fate regulators causes apoptosis and/or segregation of cell clones [see, for example, Bielmeier *et al.* (2016)]. The elimination of salivary glands in larvae by ectopic E93 may occur for similar reasons; that is, E93 may interfere with the normal developmental program of the larval salivary gland, leading secondarily to gland elimination.

By all measures, the strongest of the *Idh3b* alleles is *Idh3b^2^*. This allele is a missense change of an invariant glycine at position 278 to aspartic acid. *Idh3b^2^* may be a null allele. However, another possibility is that it encodes an interfering subunit. Mammalian IDH3 is a heterotetramer consisting of two α, one β, and one γ subunit. Biochemical reconstitution experiments show that the α and γ subunits can produce weakly active enzyme in the absence of β (Ehrlich and Colman 1983). The strength of the *Idh3b^2^* allele relative to the other alleles suggests it may encode an inactive β subunit that associates with α and γ, and blocks their residual activity. *Idh3b^1^* is an A to T change within the AG splice acceptor sequence at the 5′ boundary of exon 4. One might have expected *Idh3b^1^* to behave as a null allele, as total failure of splicing of the intron between exons 3 and 4 would result in the production of a protein consisting of only the N-terminal 61 amino acids of the 370 amino acid Idh3b protein, followed by 29 unrelated residues encoded by the unspliced intron. The relative weakness of this allele, especially when homozygous in the female germline, suggests that a cryptic splice site within exon 4 can be utilized in the mutant, or that exon 4 is entirely skipped. Splicing at the first downstream inframe AG in exon 4 would generate a transcript deleted for the first nine codons of exon 4; skipping exon 4 entirely would result in the deletion of 39 codons. *Idh3b^3^* is a missense change of an aspartic acid at position 267 to tyrosine. This position tolerates several different residues among known Idh3b sequences, but is never occupied by tyrosine in a wild-type sequence. Curiously, the relative strengths of *Idh3b^1^* and *Idh3b^3^* depend on the phenotype considered. For homozygous mutant zygotes from mutant/+ mothers, *Idh3b^1^* is stronger in its effects than *Idh3b^3^*. However, for embryos from homozygous mutant germline mothers, the reverse is true. This difference may reflect differing splicing specificities in germline- and zygotic-development, or differing Idh3b domain requirements at the two stages.

Strikingly, *Idh3b* is located within a cluster of six contiguous mitochondrial genes. These genes encode mitofilin, mitochondrial ribosomal protein L35, fumarylacetoacetase, cytochrome *c* heme lyase, and NADH dehydrogenase (ubiquinone) 42-kDa subunit, as well as Idh3b. *In situ* hybridization results reported by Baehrecke and Thummel (1995) suggest that all of these genes are contained within the 93F pupal ecdysone puff, consistent with their coregulation.

Given the central importance of the TCA cycle in metabolism, it seemed at first surprising that *Idh3b* mutants survive until the pupal stage. In humans, an *IDH3B* allele has been described that also has a surprisingly weak phenotype (Hartong *et al.* 2008). This allele causes a frameshift at codon 197, and is therefore likely a null allele. Individuals homozygous for this allele develop retinitis pigmentosa, but are otherwise normal, arguing that IDH3β is dispensable in all human tissues outside the retina. Residual IDH3 enzyme activity (presumably due to activity of IDH3α and IDH3γ) in mutant cells is only 2–5% of normal. The authors suggest that, in humans, a second mitochondrial IDH, IDH2, can fully substitute for IDH3 outside of the retina. Unlike IDH3, which is NAD dependent, IDH2 is NADP dependent, and is located within the mitochondrial intermembrane space, rather than the matrix, where the TCA cycle takes place (Chen *et al.* 2015). IDH2 is known to play an important role in the regeneration of reduced glutathione, which is utilized in the elimination of reactive oxygen species produced by the electron transport chain (Jo *et al.* 2001). However, Hartong *et al.* (2008) raise the possibility that IDH2 may also be the primary enzyme utilized in the TCA cycle. Although this hypothesis may well be correct for human cells, two observations argue that it is not true for *Drosophila*. First, we find that mitochondria from *Idh3b^2^* mutant larvae show no, or dramatically reduced, staining by dyes specific for polarized mitochondria (MitoTracker RedCMXRos and MitoTracker Deep Red), although mitochondria from wild-type control larvae are stained by these dyes. Second, we find that embryos from homozygous-mutant *Idh3b^2^* germline mothers arrest development during cleavage, with arrested embryos showing nuclear abnormalities similar to those caused by anoxia at this stage. Both of these observations suggest that the *Idh3b^2^* mutation causes an almost complete block in the TCA cycle and oxidative phosphorylation.

The survival of *Idh3b* mutants until pupariation is consistent with recent work showing that *Drosophila* larvae generate energy largely by aerobic glycolysis (Tennessen *et al.* 2011, 2014). In this mode of metabolism, which is characteristic of very rapid growth in many systems, sugars are largely converted to biomass rather than being totally oxidized, as occurs during oxidative phosphorylation [see Lunt and Vander Heiden (2011) for review]. Early embryos are dependent upon oxidative phosphorylation for energy production, and are very sensitive to anoxia (Foe and Alberts 1985; DiGregorio *et al.* 2001). However, midway through embryogenesis, genes encoding enzymes of the glycolytic pathway are globally upregulated by the *Drosophila* estrogen-related receptor (Tennessen *et al.* 2011). The glycolytic pathway then comes to dominate energy metabolism, and facilitates biomass increase during the larval stages. Consistent with early dependence on oxidative phosphorylation, embryos from homozygous-mutant *Idh3b^2^* germline mothers arrest development during cleavage. *Idh3b* mutant homozygotes from heterozygous mothers survive the embryonic period due to the presence of maternally supplied Idh3b, and then survive larval development, likely because of the metabolic shift to aerobic glycolysis at this stage. Although *Idh3b* mutants survive as larvae, their development is slowed considerably, indicating that oxidative phosphorylation continues to play an important role during normal larval development. This conclusion is supported by the polarization of mitochondria in wild-type larvae revealed by MitoTracker RedCMXRos and MitoTracker Deep Red staining.

Although the shift to aerobic glycolysis during larval development is likely the major factor allowing the survival of *Idh3b* mutants until pupariation, residual Idh3 activity provided by the Idh3 α and γ subunits may also play a role. Bypass of the TCA cycle step blocked by loss of Idh3 might also be a factor. Such bypass could occur by the action of Idh2 in the intermembrane space. As suggested by Hartong *et al.* (2008), the NADPH produced by Idh2 could be converted to NADH by nicotinamide-nucleotide transhydrogenase and transferred into the matrix. Alternatively, α-ketoglutarate produced by Idh2 could be transported into the matrix. It is also possible that citrate exported from mitochondria (as normally occurs in lipid biosynthesis) is converted to α-ketoglutarate or glutamate by cytosolic enzymes, followed by reimport of these intermediates [see Lunt and Vander Heiden (2011)].

The behavior of mitotic recombination clones homozygous for the *Idh3b* alleles is striking. In this report, we find that female germline clones homozygous for each of the *Idh3b* alleles survive and produce large numbers of normal-appearing eggs. Previously, we showed that homozygous-mutant somatic clones show no obvious growth defect, and produce normal adult cuticular structures throughout the body (Mou *et al.* 2012). The simplest interpretation is that oxidative phosphorylation is of limited importance within these cell types. Alternatively, Idh2 may be able to substitute for Idh3 in these cells, or homozygous mutant cells within clones may be rescued by their wild-type neighbors by transfer of ATP or other metabolites through gap junctions (Goldberg *et al.* 1999).

Like *Idh3b* alleles, mutations affecting another TCA cycle enzyme, Malate dehydrogenase-2 (Mdh2), arrest development after pupariation, and are defective in salivary gland death (Wang *et al.* 2010). In addition, both *Idh3b*-mutant pupae (this report) and *Mdh2*-mutant pupae (Wang *et al.* 2010) show reduced ATP levels. However, in other ways, mutants for these genes behave quite differently. *Mdh2* mutants show normal growth and produce normal-appearing puparia (Wang *et al.* 2010), whereas *Idh3b* mutations cause significant developmental delay, and show defects in puparium shape and spiracle eversion. In salivary gland cells, *Mdh2* mutants have no effect on expression of the ecdysone-induced cell death regulators *rpr*, *hid*, and *dronc*, while it has been reported that *Idh3b* mutants fail to activate transcription of these genes (Lee *et al.* 2000). Finally, *Mdh2* mutants show a normal increase in LysoTracker staining in pupal salivary glands (which signals the initiation of autophagy), while *Idh3b^2^* mutants fail to show such an increase. We do not understand why *Idh3b* mutants are more severely affected than *Mdh2* mutants. One possibility is that the TCA cycle block in *Mdh2* mutants can be partially bypassed, possibly via enzymes of the malate-aspartate shuttle.

The block in salivary gland death in *Idh3b* and *Mdh2* mutants indicates an important role for the TCA cycle and mitochondria in driving the death of larval cells during metamorphosis. As in many cell types, mitochondria in salivary gland cells undergo extensive fragmentation prior to cell death (Goyal *et al.* 2007). This fragmentation fails to occur in salivary gland cells of the *Idh3b^2^* mutant. In mammalian cells, mitochondrial fragmentation is associated with the release of apoptogenic factors such as cytochrome *c* from the mitochondrial intermembrane space [for review see Arnoult (2006)]. Although release of cytochrome *c* does not appear to promote cell death in *Drosophila* [for review see Clavier *et al.* (2016)], the failure of mitochondrial fragmentation in *Idh3b* mutants may block the release of other mitochondrial factors that promote larval cell death. Idh3b itself is a candidate for such a factor, as we find that it is released from the mitochondrial matrix after fragmentation, becoming concentrated in puncta that localize to both the cytoplasm and the nucleus. In addition, Idh3b has been reported to interact physically with the effector caspase DrICE (Guruharsha *et al.* 2011). Experiments are underway to test whether Idh3b, or other factors released from the mitochondrial matrix, play a role in promoting larval cell death.

## Supplementary Material

Supplemental material is available online at www.g3journal.org/lookup/suppl/doi:10.1534/g3.116.037366/-/DC1.
