# Supplementary figures and images for: Mutants for Drosophila Isocitrate Dehydrogenase 3b Are Defective in Mitochondrial Function and Larval Cell Death
Source: G3 (Bethesda). 2017 Jan 17;7(3):789–99. doi: 10.1534/g3.116.037366 (PMC5345709; doi:10.1534/g3.116.037366)

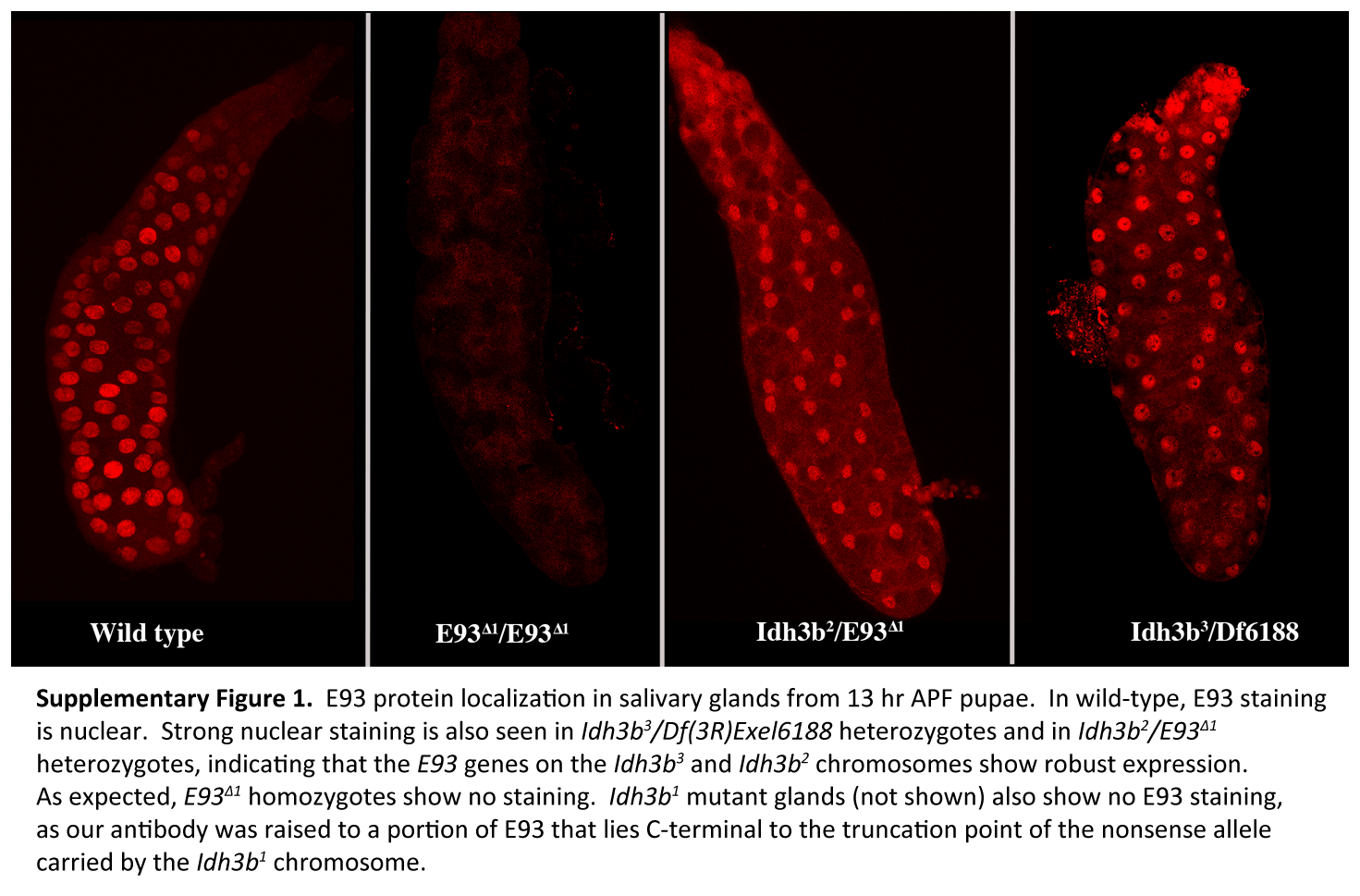

Supplement: Supplementary file 1 [file 789FigureS1.tif]

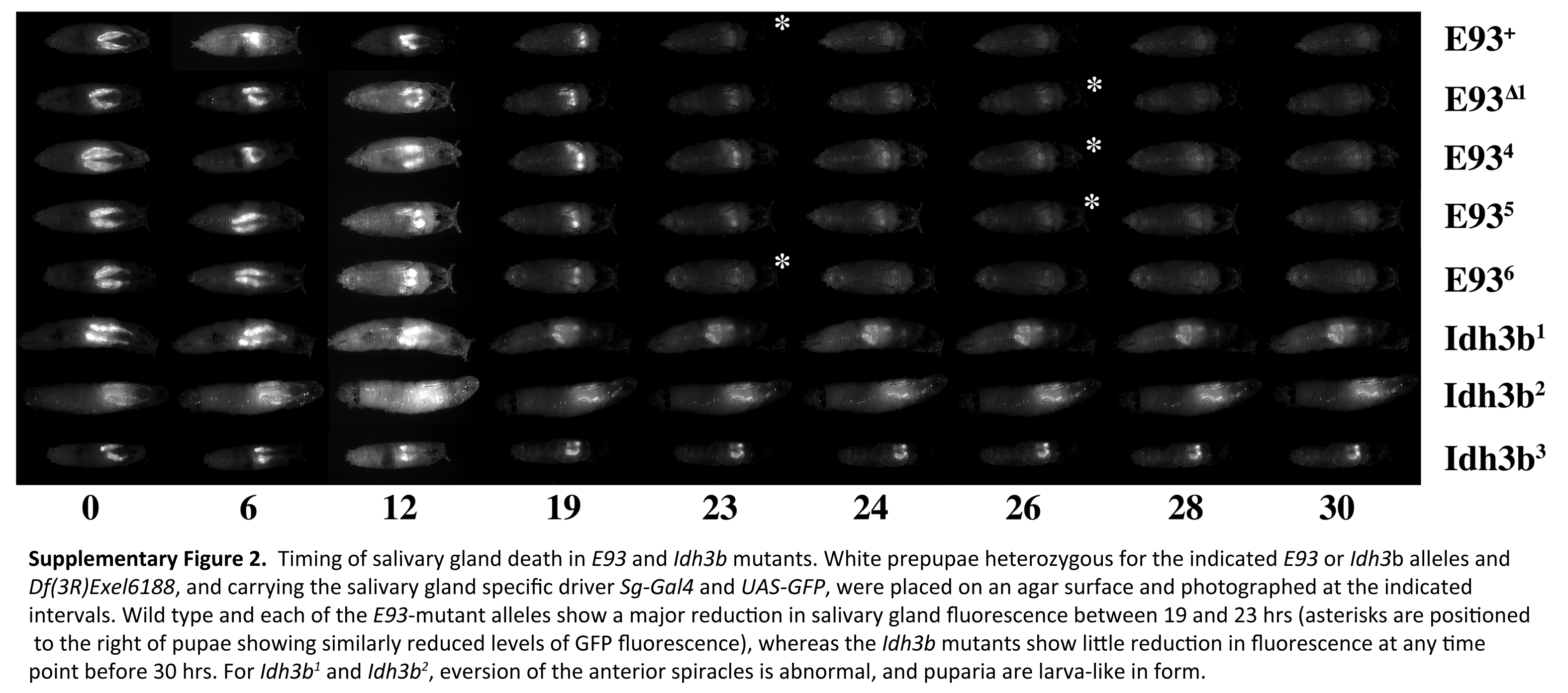

Supplement: Supplementary file 2 [file 789FigureS2.tif]

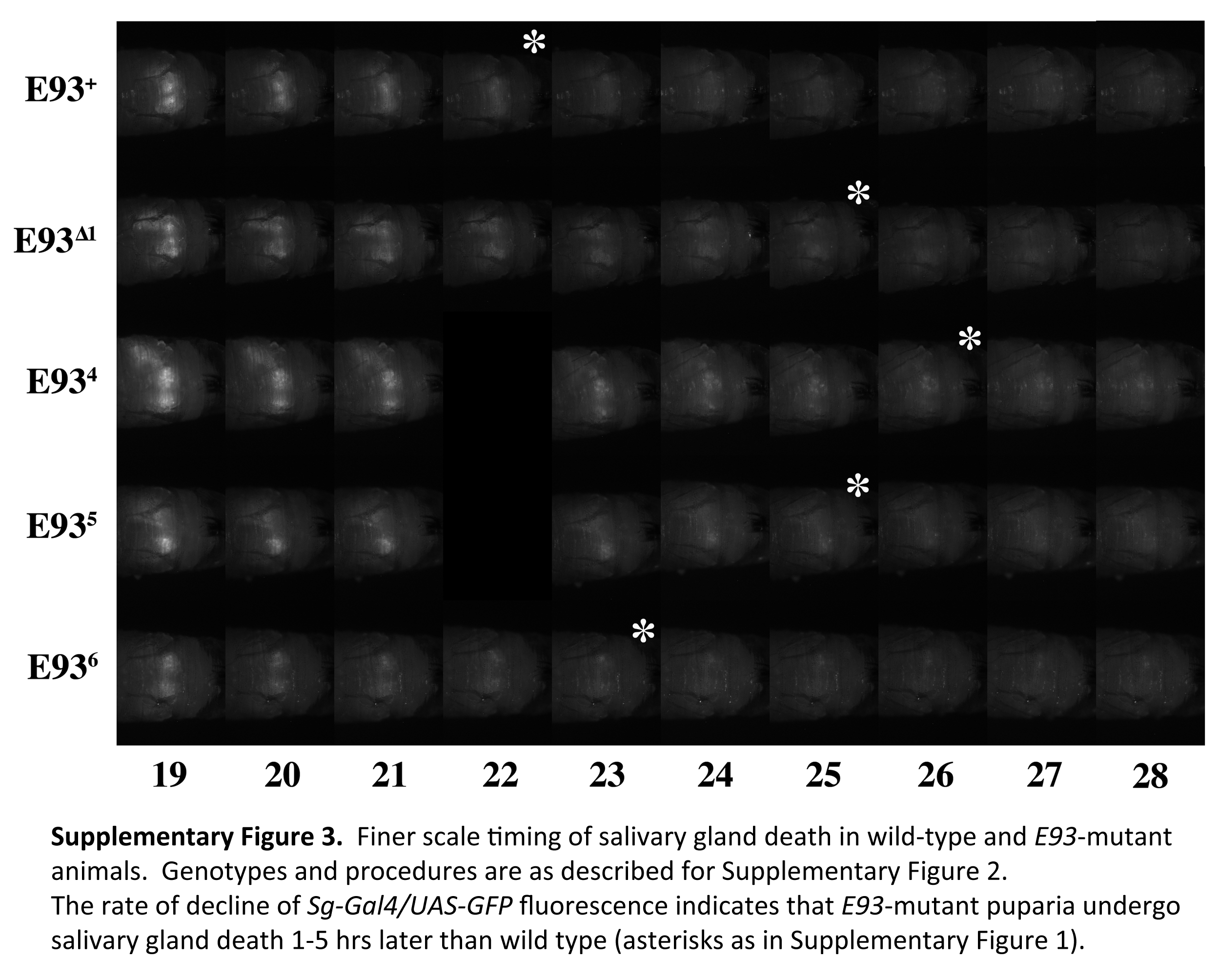

Supplement: Supplementary file 3 [file 789FigureS3.tif]
